# Elucidating adverse drug reactions and underlying molecular mechanisms of ivermectin through pharmacovigilance and multi-omics analysis

**DOI:** 10.3389/fcimb.2026.1938550

**Published:** 2026-08-26

**Authors:** Boyi Liu, Xiaoze Chen, Yishuo Wang, Hao Zhang, Xiao Liang, Xin Guan, Wenchao Zhang, Ao Han, Danna Chen

**Affiliations:** 1 The First Clinical College, Changsha Medical University, Changsha, China; 2 College of Basic Medical Sciences, Naval Medical University, Shanghai, China; 3 Department of Nuclear Medicine, First Hospital of Shanxi Medical University, Shanxi Medical University, Taiyuan, Shanxi, China; 4 Department of Basic Medical Sciences, Changsha Medical University, Changsha, Hunan, China; 5 Hunan Provincial University Key Laboratory of the Fundamental and Clinical Research on Functional Nucleic Acid, Changsha Medical University, Changsha, China

**Keywords:** adverse drug reactions, EGFR, FAERS, ivermectin, molecular docking, network toxicology, pharmacovigilance

## Abstract

**Objective:**

The comprehensive safety profile and underlying molecular mechanisms of ivermectin-associated adverse drug reactions (ADRs) remain to be fully elucidated.

**Methods:**

We integrated pharmacovigilance data from the FDA Adverse Event Reporting System (FAERS) with network toxicology and transcriptomic validation. Disproportionality analyses were conducted on 1,421 ivermectin-related reports to detect signals at the System Organ Class (SOC) and Preferred Term (PT) levels. Network-based approaches, including protein-protein interaction (PPI) analysis and molecular docking, were employed to identify core toxicity targets, followed by ADMET property prediction.

**Results:**

Significant safety signals emerged for Eye disorders, Nervous system disorders, and General disorders. Frequently reported PTs included asthenia, headache, and pyrexia, alongside notable serious signals such as encephalopathy (ROR = 24.1) and toxic encephalopathy (ROR = 16.18). Network toxicology identified five core targets shared across key SOCs: EGFR, ERBB2, TGFB1, PIK3CA, and HSPG2. KEGG enrichment analysis highlighted pathways related to parasitic diseases, leukocyte migration, and endocrine regulation. Molecular docking confirmed high binding affinity between ivermectin components and EGFR (≤ -8.7 kcal/mol). ADMET predictions indicated elevated risks for genotoxicity, ototoxicity, and skin sensitization.

**Conclusion:**

Ivermectin-associated ADRs manifest across multiple organ systems, which are potentially associated with the modulation of predicted candidate targets including EGFR, ERBB2, TGFB1, PIK3CA, and HSPG2.

## 1 Introduction

Ivermectin is a broad-spectrum antiparasitic drug that has been widely used in the treatment of helminth infections since its market launch (Abu-Zaid et al., 2025; Seyyedabadi et al., 2025; Yilmaz et al., 2026). In recent years, its potential off-label uses, such as antiviral, anti-inflammatory, and anti-tumor effects, have gradually attracted attention, further expanding its clinical application scope. However, with the increase in the number of users, reports of ivermectin-associated adverse drug reactions (ADRs) have increased year by year, involving multiple organ systems such as the eyes, nervous system, and skin. In severe cases, it can cause serious adverse reactions such as encephalopathy and toxic encephalopathy, threatening the safety of patients’ medication. At present, most studies on ivermectin-associated ADRs focus on clinical reports of single adverse reactions, and the comprehensive safety profile and underlying molecular mechanisms have not been fully elucidated, which is difficult to meet the needs of clinical rational drug use and risk prevention and control.

The monitoring and mechanism research of adverse drug reactions are the core links to ensure clinical rational drug use. The FDA Adverse Event Reporting System (FAERS), established by the U.S. Food and Drug Administration, is one of the world’s largest spontaneous adverse drug reaction monitoring databases (Nakano et al., 2026; Yan et al., 2026). It systematically collects adverse drug event reports worldwide and provides abundant data support for post-marketing drug safety signal mining. Through methods such as disproportionality analysis, potential safety signals related to drugs can be quickly screened from the FAERS database, the distribution characteristics and risk intensity of ADRs can be clarified, and clinical guidance can be provided for subsequent mechanism research.

As an important branch of systems biology, network toxicology integrates the research ideas of network pharmacology and toxicology. It can predict the potential toxic targets of drugs by integrating multiple database resources, construct target-pathway networks, thereby systematically analyzing the molecular mechanisms of drug-induced multi-organ toxicity, and breaking the research limitation of traditional toxicology of “single target-single effect”. In recent years, network toxicology has been widely used in the mechanism research of adverse drug reactions. For example, in studies on metformin-induced renal injury and atorvastatin-induced muscle injury, core toxic targets have been successfully screened by this method, providing important clues for the elucidation of molecular mechanisms (An et al., 2026; Tang et al., 2026).

As the core method of computer-aided drug design, molecular docking technology can simulate the interaction between drug small molecules and target proteins, quantify the binding affinity between them through binding energy, verify the direct interaction relationship between drugs and targets, and provide molecular-level evidence for the core targets predicted by network toxicology. Existing studies have shown that combining clinical signal mining from the FAERS database with network toxicology and molecular docking technology can realize a closed-loop research of “clinical phenomenon-target prediction-molecular verification”, effectively explore the potential molecular mechanisms of adverse drug reactions, and provide a scientific basis for clinical medication safety (He et al., 2026; Xu et al., 2026).

Based on this, this study adopted an integrated research method. Taking the FAERS database as the basis, disproportionality analysis was used to mine the safety signals of ivermectin-associated ADRs, and their distribution characteristics and risk intensity in different organ systems were clarified; network toxicology was combined to screen the core toxic targets of ivermectin and analyze the signaling pathways involved; molecular docking was used to verify the binding affinity between ivermectin and core targets, and transcriptomic data was used to verify the expression regulation pattern of targets; at the same time, ADMET property prediction was combined to comprehensively evaluate the toxicity risk of ivermectin. This study aims to systematically elucidate the safety profile and potential molecular mechanisms of ivermectin-associated ADRs, fill the current research gaps, and provide important theoretical basis and practical guidance for the clinical rational use, risk monitoring, and toxicity intervention of ivermectin.

## 2 Materials and methods

### 2.1 Data source

The FDA Adverse Event Reporting System (FAERS), maintained by the U.S. Food and Drug Administration, serves as a critical pharmacovigilance tool for post-marketing surveillance of pharmaceuticals and therapeutic biological products. This comprehensive database systematically collects and analyzes spontaneous reports of adverse drug events and medication errors. Consequently, it enables the identification and evaluation of potential drug safety signals. The FAERS database is updated quarterly, and all datasets are publicly accessible through the FDA’s open data portal.

### 2.2 Data extraction and processing

The FAERS database architecture comprises seven distinct data tables, each serving specific pharmacovigilance functions: (1) DEMO (patient demographic data), (2) DRUG (drug-related data), (3) REAC (adverse event reports), (4) OUTC (patient outcomes), (5) RPSR (report source information), (6) THER (drug therapy data), and (7) INDI (indication for use). Within the drug table, medications are systematically categorized into four distinct roles: Primary Suspect (PS), Secondary Suspect (SS), Concomitant (C), and Interacting (I). Severe adverse outcomes are classified according to FDA-defined criteria, including: death (DE), life-threatening conditions (LT), hospitalization (HO), disability (DS), congenital anomaly (CA), and other medically important events (OT). Case identification was conducted by querying drug names within the drug table, followed by filtering to include only Primary Suspect (PS) cases. Adverse event terminology was standardized using the Medical Dictionary for Regulatory Activities (MedDRA), which employs a five-level hierarchical classification system: System Organ Class (SOC), High-Level Group Term (HLGT), High-Level Term (HLT), Preferred Term (PT), and Lowest Level Term (LLT).

### 2.3 Data preprocessing and quality control

This study employed the FDA-recommended data extraction protocol to retrieve all case reports involving Ivermectin and submitted by physicians between the first quarter of 2004 (Q1 2004) and the fourth quarter of 2025 (Q4 2025). To ensure data integrity and minimize potential biases in disproportionality analysis, stringent quality control measures were implemented, including the systematic removal of duplicate reports using an FDA-recognized deduplication algorithm.

Specifically, following the FDA guidelines for duplicate removal, we selected the PRIMARYID, CASEID, and FDA_DT fields from the DEMO table. Reports were sorted sequentially by CASEID, FDA_DT, and PRIMARYID. For reports sharing identical CASEIDs, the record with the maximum FDA_DT value was retained. In cases where both CASEID and FDA_DT were identical, the report with the highest PRIMARYID value was retained.

### 2.4 Disproportionality analysis

Disproportionality analysis represents a fundamental methodology in pharmacovigilance and drug safety monitoring, designed to identify potential safety signals by detecting statistical imbalances in adverse drug reaction (ADR) reporting patterns. The analytical framework encompasses several established metrics, including the Reporting Odds Ratio (ROR), the Bayesian Confidence Propagation Neural Network (BCPNN), the Proportional Reporting Ratio (PRR), and the Multi-item Gamma Poisson Shrinker (MGPS).

In the current analysis, we specifically implemented the Reporting Odds Ratio (ROR) and BCPNN methods to detect adverse reaction signals, leveraging the System Organ Class (SOC) and Preferred Term (PT) classifications of the target drug. **Supplementary Table 1** provides a detailed 2×2 contingency matrix, while **Supplementary Table 2** specifies the concrete parameters of the two primary signal detection algorithms.

To mitigate potential confounding by indication—a common limitation in spontaneous reporting systems—an active comparator analysis was conducted using albendazole, another widely prescribed broad-spectrum antiparasitic agent. Adverse reaction signals for albendazole were evaluated utilizing identical disproportionality algorithms and subsequently compared against the primary suspect cases of ivermectin. This approach was employed to rigorously differentiate drug-specific safety profiles from disease-related background risks.

### 2.5 Network toxicological analysis

Network toxicology is a systems biology-based approach that leverages network pharmacology techniques to rapidly predict the mechanisms of drug toxicity. The chemical structures and SMILES notations of the two main components of ivermectin (Ivermectin B1a and Ivermectin B1b) were obtained from the PubChem database. Predicted targets for Ivermectin B1a and Ivermectin B1b were retrieved from the TOXRIC, Similarity Ensemble Approach, ChEMBL, Comparative Toxicogenomics Database, TargetNet, GeneCards, and SuperPRED databases. Additionally, targets associated with key site-of-concern (SOC) were collected using the GeneCards database. Intersection targets between the predicted ivermectin targets and SOC-related targets were identified using Venn diagrams, and these overlapping targets were considered potential toxicity targets of ivermectin relevant to the SOC. A protein-protein interaction (PPI) network of the potential toxicity targets was constructed using the STRING database and Cytoscape software (version 3.10.3) to identify key targets. Finally, Kyoto Encyclopedia of Genes and Genomes (KEGG) and Gene Ontology (GO) enrichment analyses were performed on the potential toxicity targets of key SOCs using the clusterProfiler R package, followed by visualization.

### 2.6 Transcriptome data analysis

To validate the expression regulation patterns of core targets, the transcriptomic dataset GSE201404 was obtained from the GEO database. This dataset comprises a total of 10 samples from two human esophageal squamous cell carcinoma cell lines (KYSE30 and KYSE70) before and after ivermectin treatment. Gene expression levels were quantified using FPKM (Fragments Per Kilobase of transcript per Million mapped reads) values, and visualization of differentially expressed genes was performed using the pheatmap package in R language.

### 2.7 Molecular docking

Virtual molecular docking was performed following previously established methods to assess the affinity between the drug and key target proteins. The molecular structures of the drug and the target proteins were downloaded from the PubChem database and the RCSB PDB database, respectively. Ligand compounds were subjected to energy minimization using Chem3D software and subsequently converted into mol2 format. Water molecules, metal ions, and ligands bound to the protein macromolecules were removed, and the resulting structures were converted into PDB files. Subsequently, semi-flexible molecular docking (in which the protein macromolecule remained rigid while the small-molecule ligand was treated as flexible) was performed using AutoDock software based on the Lamarckian genetic algorithm. Binding energy was used to evaluate the affinity between the ligand (drug) and the receptor (target protein). Visualization was carried out using PyMOL software.

### 2.8 ADMET property prediction

The ADMETlab 3.0 platform was employed to systematically predict the absorption, distribution, metabolism, excretion, and toxicity (ADMET) of the two components of ivermectin. The evaluated parameters focused on water solubility (LogS), lipophilicity (LogP/LogD7.4), hepatotoxicity (DILI), hERG cardiotoxicity, genotoxicity (AMES test), carcinogenicity, nephrotoxicity, neurotoxicity, ototoxicity, skin sensitization, cytotoxicity (A549, HEK293), and acute toxicity (LC50, IGC50). The prediction results are expressed as risk probability values ranging from 0 to 1, where values closer to 1 indicate a higher risk of the respective toxicity. The prediction results are expressed as risk probability values ranging from 0 to 1. In our evaluation, probability values approaching 1.0 (specifically ≥0.8) were defined as indicative of a high theoretical risk for the respective toxicity end-points.

## 3 Result

### 3.1 Descriptive analysis

**Figure 1:**
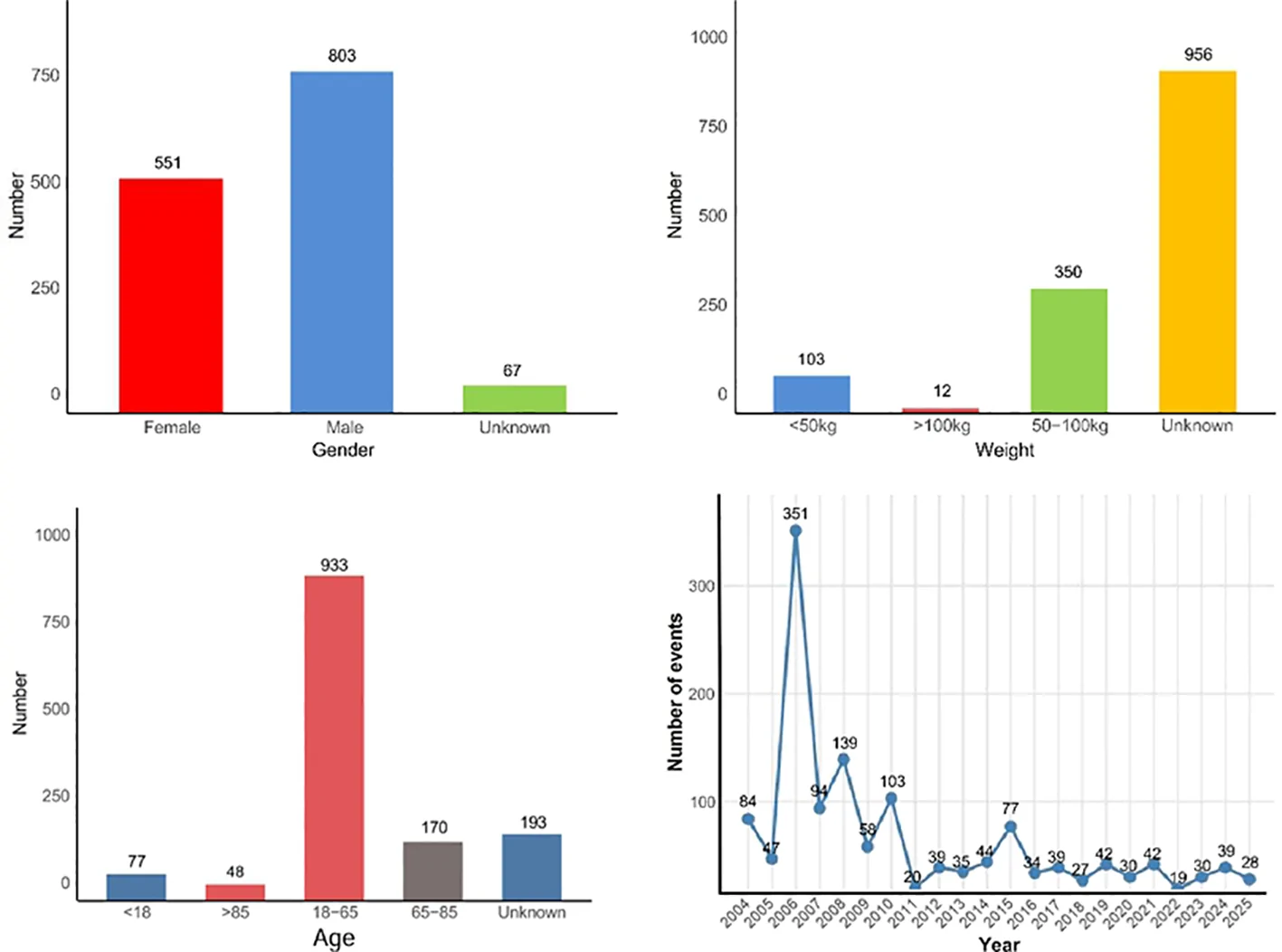
Distribution of baseline clinical characteristics of cases with ivermectin-related adverse event reports.

We analyzed adverse event (AE) reports associated with ivermectin (n=1421). **Figure 1** summarizes the baseline clinical characteristics of the reported patients. The proportion of reports involving male patients (n=551) was lower than that of female patients (n=803). The most frequently reported body weight range was 50–100 kg (n=350), with a substantial number of cases missing weight data (n=956). Regarding age, the most commonly reported group for ivermectin was 18–65 years (n=933), followed by elderly patients aged ≥65 years (n=274). A peak was observed in 2006, after which the number of reports remained stable.

### 3.2 Signal detection based on SOC levels

**Figure 2:**
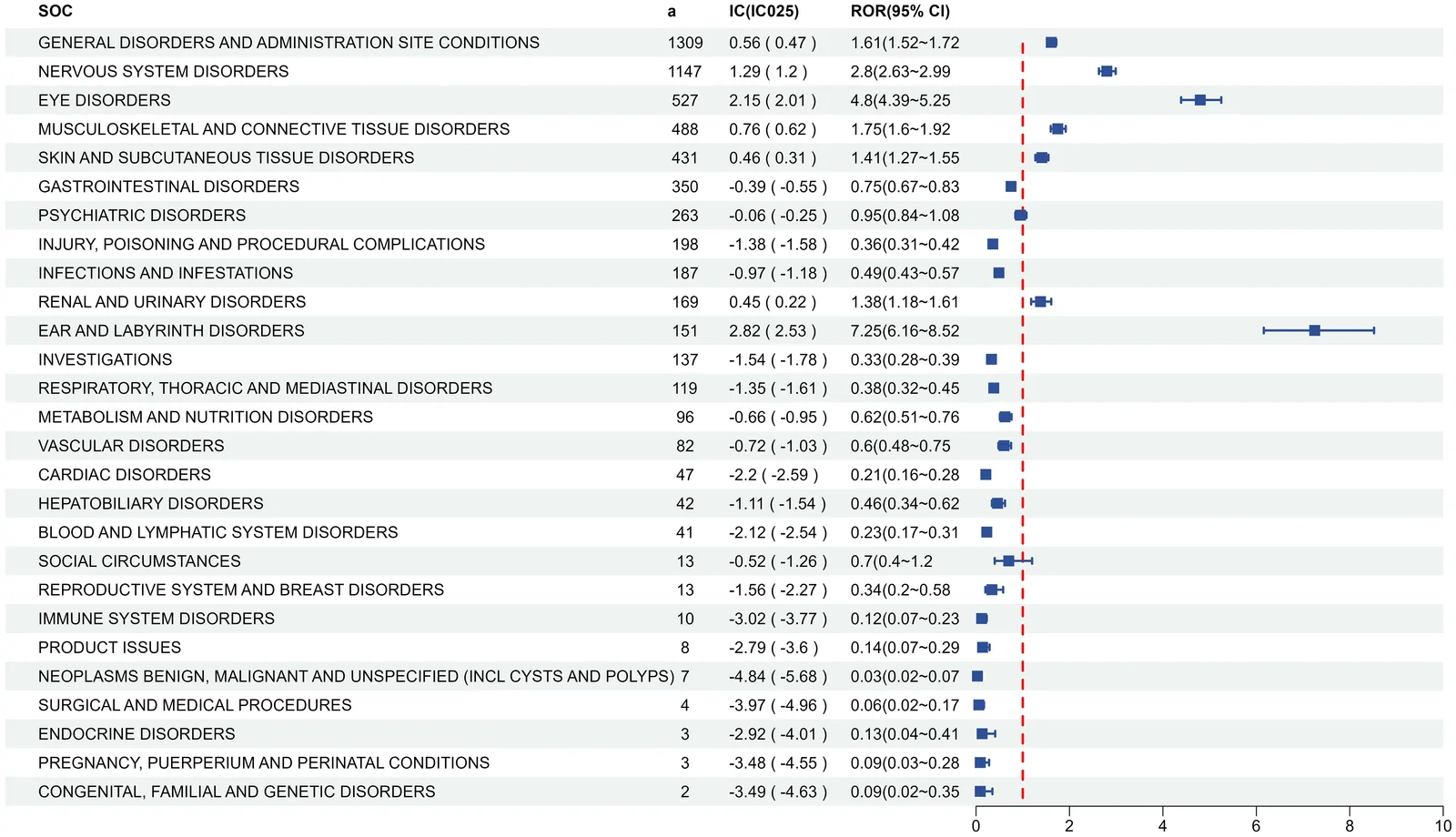
Number of adverse event reports and disproportionality signal strength at the System Organ Class (SOC) level for ivermectin.

**Figure 2** presents the signal intensity of adverse reactions at the System Organ Class (SOC) level for ivermectin. For ivermectin, the categories that met both algorithmic criteria (ROR and BCPBN) were eye disorders (n=527), nervous system disorders (n=1147), musculoskeletal and connective tissue disorders (n=488), general disorders and administration site conditions (n=1309), ear and labyrinth disorders (n=151), renal and urinary disorders (n=169), and skin and subcutaneous tissue disorders (n=431). The three SOCs with the highest frequency of adverse reactions were general disorders and administration site conditions (n=1309), eye disorders (n=527), and nervous system disorders (n=1147).

### 3.3 Signal detection based on PT levels

**Figure 3:**
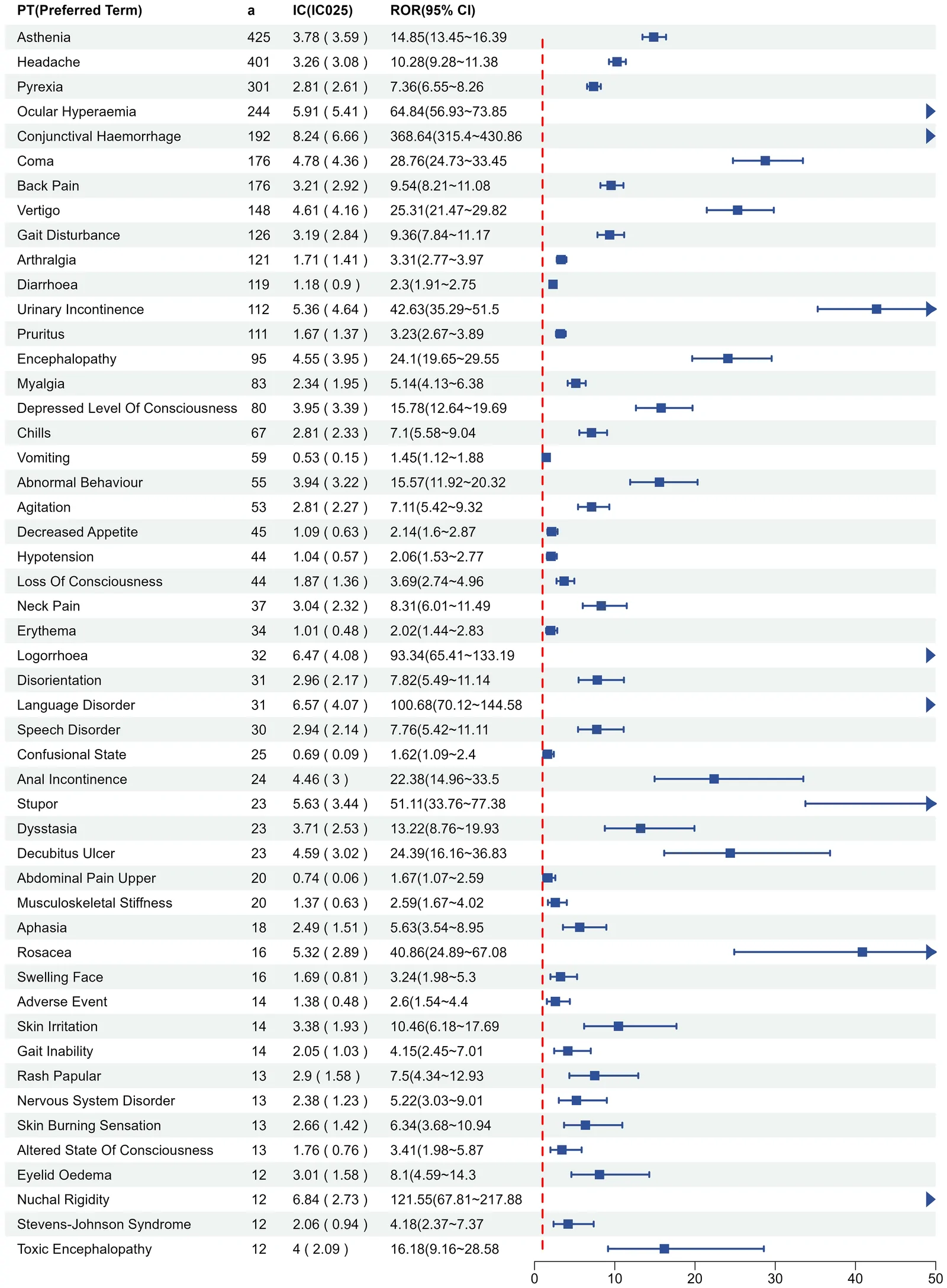
Signal detection results at the Preferred Term (PT) level for ivermectin-associated adverse events (top 50). PTs meeting the positive criteria of both ROR and BCPNN algorithms are displayed.

As shown in **Figure 3**, in the analysis at the Preferred Term (PT) level, we first screened PTs that met the criteria of both algorithms (ROR and BPCPBN), subsequently excluded terms unrelated to adverse drug reactions, and finally identified statistically significant signals. For ivermectin, common adverse reactions included asthenia (n=425), headache (n=401), and pyrexia (n=301). Common cutaneous toxicities included pruritus (n=111, ROR = 3.23, 95% CI = 2.67–3.89; IC = 1.67, IC025 = 1.37), erythema (n=34, ROR = 2.02, 95% CI = 1.44–2.83; IC = 1.01, IC025 = 0.48), and hemorrhoidal ulcer (n=23, ROR = 2.02, 95% CI = 16.16–36.83; IC = 4.59, IC025 = 3.02). Additionally, several potential adverse reactions with varying signal intensities were identified, including encephalopathy (n=95, ROR = 24.1, 95% CI = 19.65–29.55; IC = 4.55, IC025 = 3.95), hemiplegia (n=5, ROR = 4.42, 95% CI = 1.84–10.64; IC = 2.14, IC025 = 0.31), and toxic encephalopathy (n=12, ROR = 16.18, 95% CI = 9.16–28.58; IC = 4, IC025 = 2.09). These potential signals warrant attention in clinical practice. Detailed PTs are presented in **Supplementary Table 3**.

### 3.4 Subgroup analysis methodology

This study investigated potential sex differences in adverse reactions to ivermectin through subgroup analysis by sex. To evaluate the statistical significance of these sex-specific differences, we compared the 95% confidence intervals (CIs) of the reporting odds ratios (RORs) between the subgroups; non-overlapping 95% CIs were considered indicative of a statistically significant difference. According to the supplementary table, the most common adverse reactions in both male and female patients receiving ivermectin were asthenia, headache, and pyrexia. notably, asthenia occurred most frequently in males (n=341), whereas headache was more common in female patients (n=173). Among these, adverse reactions such as monoparesis, mutism, and meningitis were reported only in males, while distinct adverse reactions including alopecia, pain in extremity, and application site conditions occurred exclusively in female patients. These findings suggest that sex-specific differences in adverse reactions should be carefully considered when using ivermectin in clinical practice, and corresponding targeted monitoring and preventive measures should be implemented. Detailed PTs are presented in **Supplementary Tables 4**, **5**.

### 3.5 Active comparator analysis with albendazole

To further validate that the observed safety signals were intrinsic to ivermectin rather than artifacts of the underlying parasitic indications, a comparative pharmacovigilance analysis was performed using albendazole as an active class-matched control.

As detailed in the supplementary data, while both antiparasitic drugs shared general adverse event signals such as pyrexia (Ivermectin ROR = 7.36 vs. Albendazole ROR = 4.10) and asthenia (Ivermectin ROR = 14.85 vs. Albendazole ROR = 2.69), ivermectin exhibited notably higher signal intensities. Crucially, while severe neurological signals including encephalopathy and toxic encephalopathy were detected in both cohorts, ivermectin demonstrated a substantially higher risk magnitude (Encephalopathy ROR = 24.1 vs. 10.13; Toxic Encephalopathy ROR = 16.18 vs. 13.25). Relevant data are shown in **Supplementary Table 6**.

Furthermore, several prominent signals uniquely characterizing ivermectin’s safety profile in our primary analysis—such as headache, ocular hyperaemia, erythema, and pruritus—did not emerge as positive signals in the albendazole dataset. This distinct divergence confirms that the pronounced ocular, neurological, and cutaneous toxicities are specifically driven by ivermectin’s unique pharmacological mechanisms and tissue distribution, thereby effectively excluding confounding by indication.

### 3.6 Physicochemical properties and toxicity assessment of ivermectin

**Figure 4:**
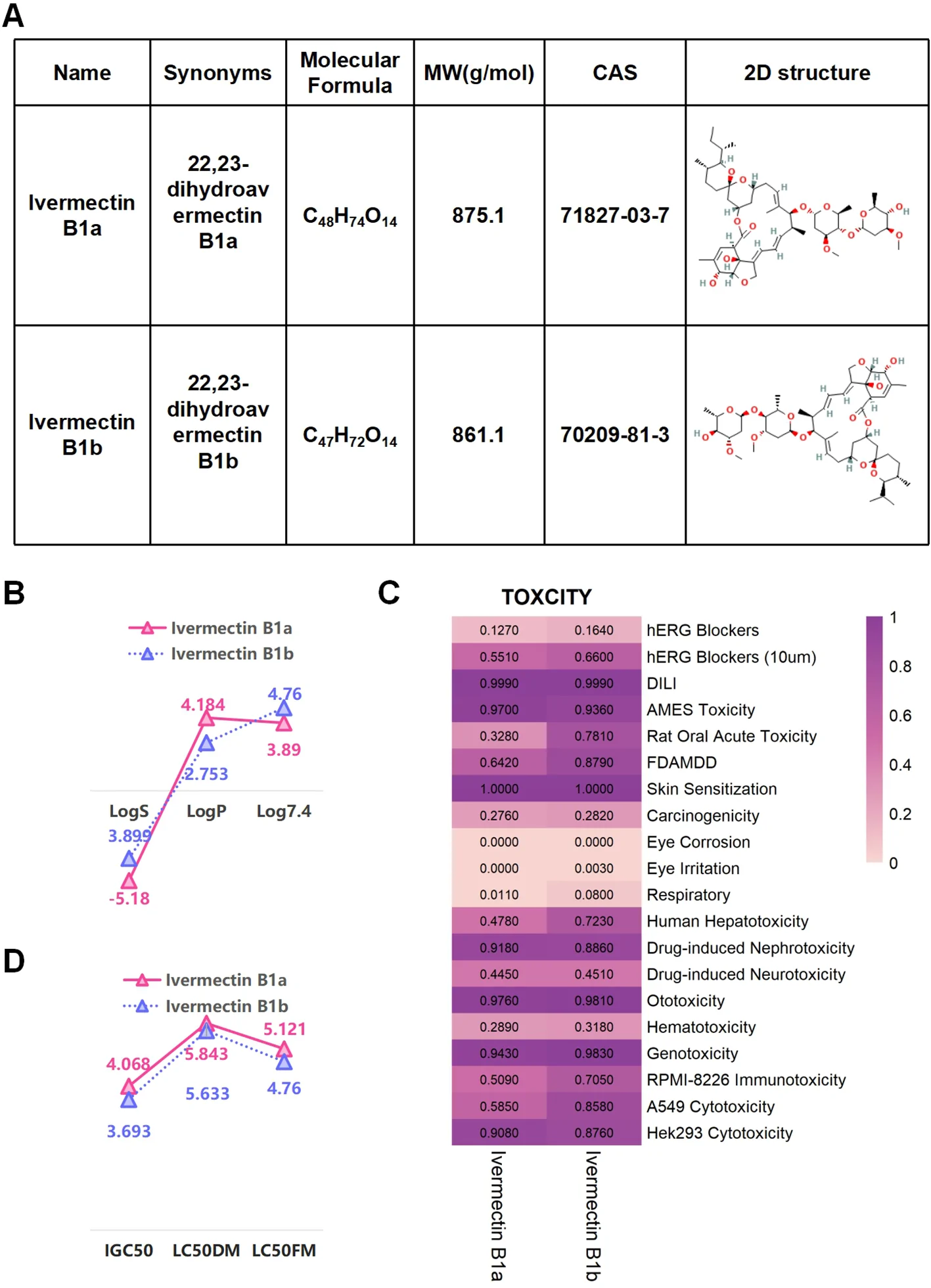
Physicochemical properties and toxicity assessment of Ivermectin. **(A)** Physicochemical properties of Ivermectin compounds obtained from the PubChem database. **(B)** Predicted logS, logP, and logD7.4 values. **(C)** Predicted toxicity, represented as the probability of being toxic (ranging from 0 to 1). **(D)** Predicted IGC50, LC50DM, and LC50FM values. logS: logarithm of the aqueous solubility; logP: logarithm of the partition coefficient; logD7.4: logarithm of the distribution coefficient at pH 7.4. IGC50: 48-hour Tetrahymena pyriformis Inhibitory Growth Concentration 50%; LC50DM: 48-hour Daphnia magna Lethal Concentration 50%; LC50FM: 96-hour Fathead minnow Lethal Concentration 50%.

To comprehensively evaluate the physicochemical properties, pharmacokinetic characteristics, and toxicity risks of the two components of ivermectin (Ivermectin B1a and Ivermectin B1b), systematic predictions were performed in this study. The Synonyms, molecular formulas, molecular weights, CAS numbers, and 2D structures of these two compounds were obtained from the PubChem database (**Figure 4A**). Regarding physicochemical properties, both components exhibited high lipophilicity (LogP > 2.7) and moderate to good water solubility (LogS ranging from -5.18 to 4.07), consistent with their favorable oral absorption characteristics (**Figure 4B**). In terms of toxicity risks, both components showed high predicted probabilities for genotoxicity (AMES toxicity: 0.9990 for both; genotoxicity: 0.9430 for B1a, 0.9830 for B1b), ototoxicity (0.9760 for B1a, 0.9810 for B1b), skin sensitization (1.0000 for both), and nephrotoxicity (0.9180 for B1a, 0.8860 for B1b). Moderate to high risks were also observed for hepatotoxicity (0.4780 for B1a, 0.7230 for B1b), cardiotoxicity (hERG blockade: 0.5510 for B1a, 0.6600 for B1b), and cytotoxicity (A549 and HEK293 cells: 0.9080 for B1a, 0.8760 for B1b). In contrast, risks for eye irritation (0.0000 for B1a, 0.0030 for B1b) and respiratory toxicity (0.0110 for B1a, 0.0800 for B1b) were low (**Figure 4C**). Regarding ecotoxicity and acute toxicity, the IGC50 values were 5.121 μM (B1a) and 5.843 μM (B1b), the LC50DM values were 5.633 μM (B1a) and 3.693 μM (B1b), and the LC50FM values were 4.76 μM for both, suggesting that Ivermectin B1b may pose a higher toxicity risk in acute exposure scenarios (**Figure 4D**). In summary, while the risk values for most toxicity endpoints were similar between the two components, Ivermectin B1b exhibited slightly higher risks than B1a in terms of hepatotoxicity, immunotoxicity, hERG blockade, and acute toxicity.

### 3.7 PPI network analysis of toxicity targets of ivermectin key SOCs

**Figure 5:**
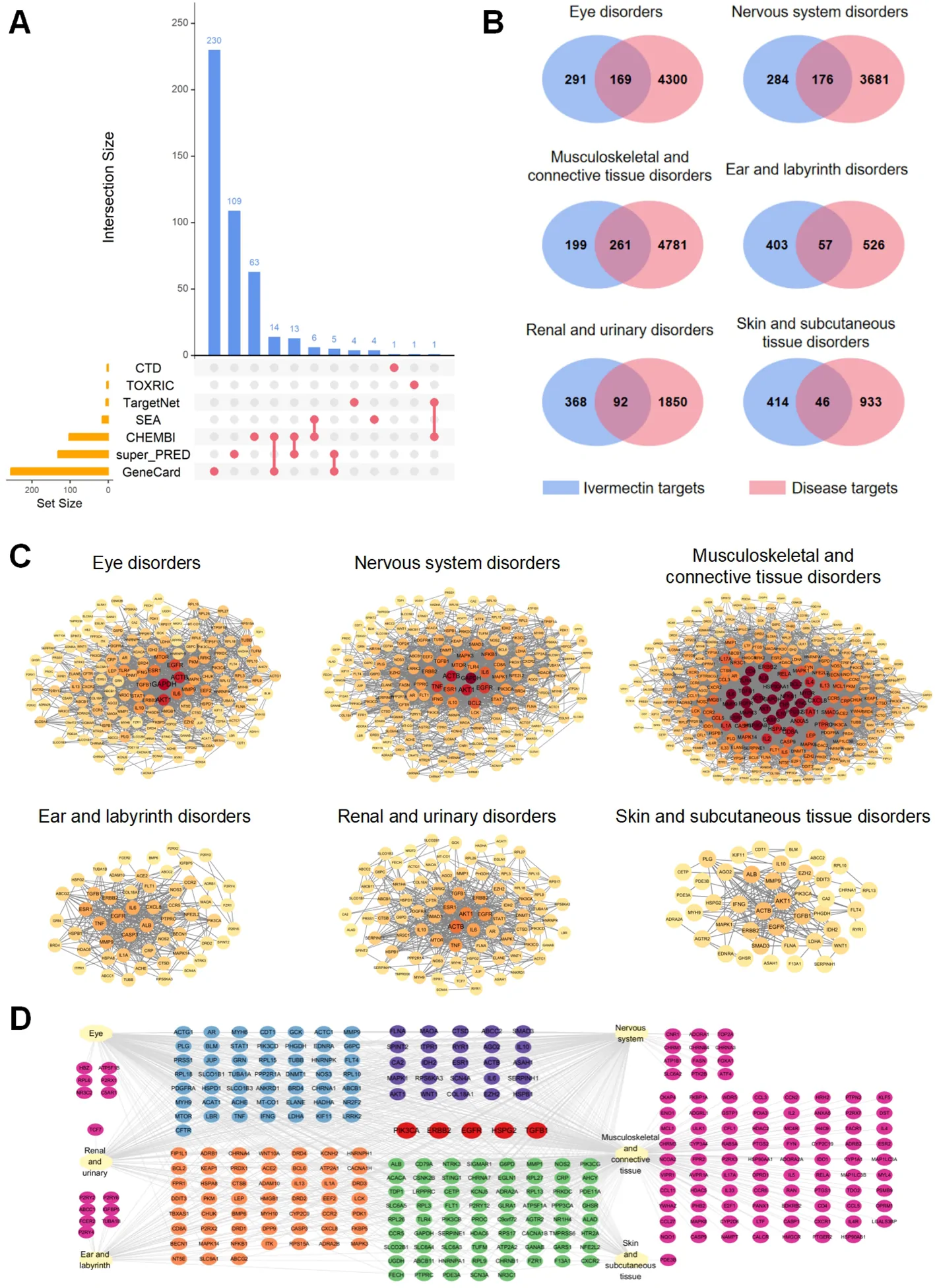
PPI networks of potential toxic targets of Ivermectin. **(A)** Sources of predicted targets of Ivermectin. **(B)** Venn diagram of prediction targets and key SOCs. **(C)** PPI network map of common targets and six key SOCs. **(D)** Network Venn diagram of potential toxicity targets of six key SOCs.

To investigate the toxicological mechanisms underlying adverse drug events (ADEs) associated with ivermectin, SOCs with a PT count greater than 3 and an ADE report count greater than 10 were selected as key SOCs, and network toxicology was employed to analyze their toxicological mechanisms. Since “Infections and infestations” and “General disorders and administration site conditions” are not specific to systemic organ diseases, they were excluded from the toxicological mechanism analysis. A total of 460 predicted targets of ivermectin were collected from seven databases, with some targets being co-predicted by multiple databases (**Figure 5A**). Relevant targets for the six key SOCs were collected from the GeneCards database, and their intersection with ivermectin targets was identified using Venn diagrams; these overlapping targets were considered potential toxicity targets involved in the occurrence of ivermectin-related ADEs (**Figure 5B**). Subsequently, a protein-protein interaction (PPI) network was constructed using the STRING database and Cytoscape software. Topological analysis revealed that the six SOCs shared similar key nodes, including EGFR, ERBB2, and TGFB1 (**Figure 5C**). The network diagram showed that the six SOCs had many common potential toxicity targets, among which EGFR, ERBB2, TGFB1, PIK3CA, and HSPG2 were shared by all SOCs. Additionally, “Eye disorders”, “Renal and urinary disorders”, “Nervous system disorders”, and “Musculoskeletal and connective tissue disorders” together shared 33 targets (**Figure 5D**). Furthermore, certain SOC-specific targets were also identified, such as 79 targets uniquely associated with “Musculoskeletal and connective tissue disorders” and 12 targets uniquely associated with “Nervous system disorders”.

### 3.8 GO and KEGG enrichment analysis of potential toxic targets of key SOCs of ivermectin

**Figure 6:**
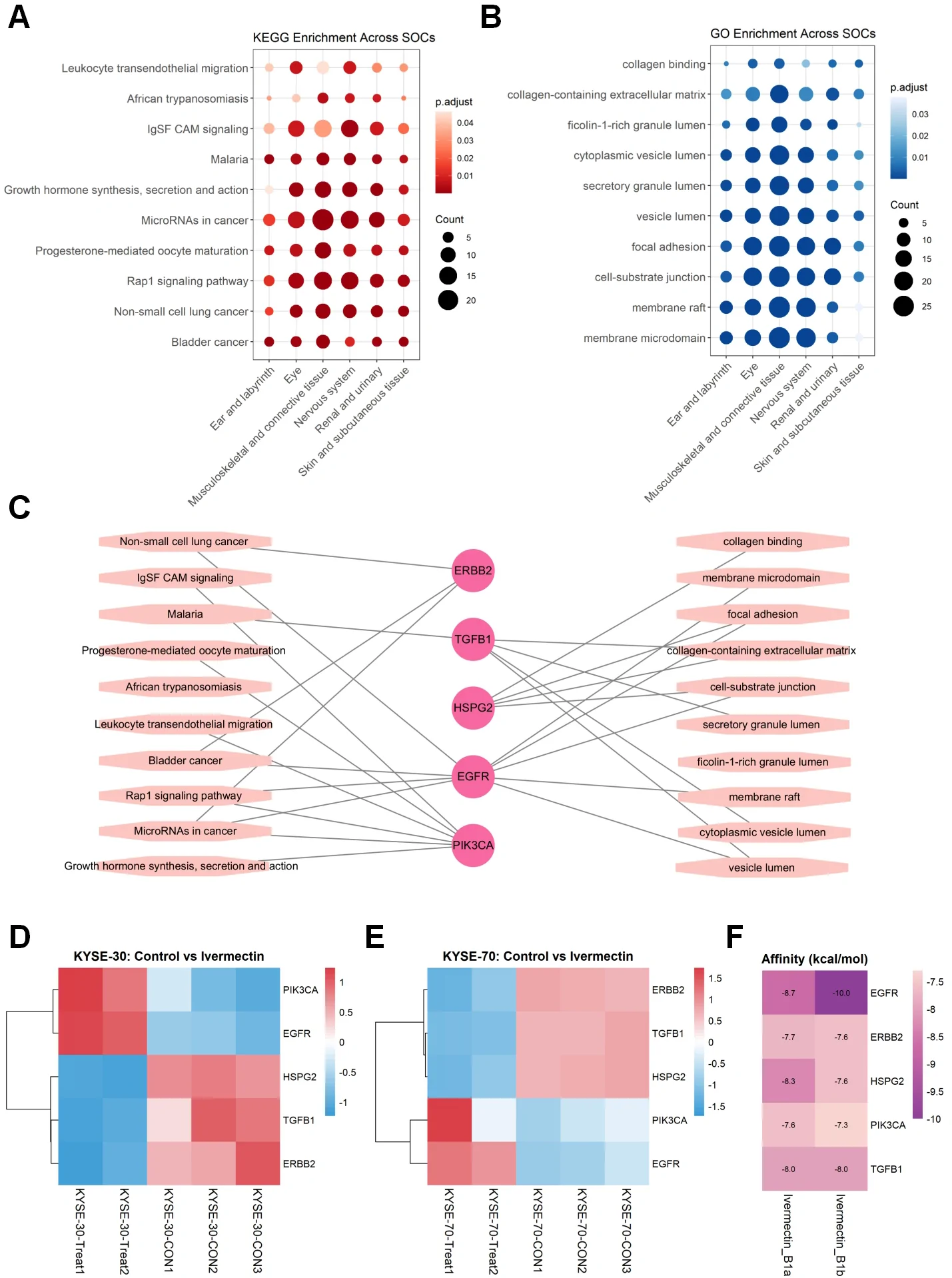
Mechanism analysis of potential toxic targets of key SOCs of Ivermectin. GO functional **(A)** and KEGG pathway **(B)** enrichment analysis of potential toxic targets of six key SOCs. **(C)** pathway-target network. **(D, E)** Heatmap showing the expression of core targets after Ivermectin intervention in KYSE-30 **(D)** and KYSE-70 **(E)**. **(F)** Heatmap of binding energy between Ivermectin and proteins expressed by key toxicity genes.

To further investigate the role of toxicity targets of key SOCs of ivermectin in regulating cellular life processes, GO and KEGG enrichment analyses were performed on the toxicity targets of each SOC. In the KEGG pathway enrichment analysis, among the top 100 significant pathways for each of the six key SOCs, a total of 10 pathways were shared among them. These mainly included parasitic disease pathways such as African trypanosomiasis, immune/inflammation-related pathways such as leukocyte transendothelial migration, endocrine-related pathways such as growth hormone synthesis, secretion and action, as well as several cancer-related pathways (**Figure 6A**). These findings provide clues for understanding how ivermectin may interfere with immune, endocrine, and cell growth-related signaling pathways across multiple SOCs, thereby inducing systemic toxicity. For the GO enrichment analysis of toxicity targets of the six key SOCs, the study further intersected the top 150 most significant terms in each of the three categories—cellular component (CC), biological process (BP), and molecular function (MF)—for each SOC, yielding a total of 10 shared GO terms. These included nine cellular component terms (e.g., collagen-containing extracellular matrix, ficolin-1-rich granule lumen, cytoplasmic vesicle lumen, secretory granule lumen, vesicle lumen, focal adhesion, cell-substrate junction, membrane raft, membrane microdomain) and one molecular function term (collagen binding) (**Figure 6B**). This set of results, at the level of cellular structure and microenvironment, echoes the functional implications of cell adhesion, migration, and immune regulation suggested by the KEGG enrichment analysis, further revealing that ivermectin may participate in mediating its multi-organ toxic effects by influencing the extracellular matrix, adhesion structures, and membrane signaling microdomains.

### 3.9 Construction of target- pathway network and identification of key toxicity target

EGFR, ERBB2, TGFB1, PIK3CA, and HSPG2 were shared targets across all SOCs and were therefore considered predicted candidate targets in the toxicological effects of ivermectin. To elucidate the specific associations between these five core SOC targets and functional pathways, a “target-enrichment term” association network was constructed in this study. The results showed that PIK3CA, EGFR, and ERBB2 were primarily associated with KEGG pathways related to cell proliferation, immune migration, and endocrine regulation (e.g., Rap1 signaling, leukocyte migration, cancer pathways), constituting a signal transduction/growth regulation module. In contrast, TGFB1 and HSPG2 were mainly associated with GO terms related to extracellular matrix structure, focal adhesions, and immune granules (e.g., collagen-containing ECM, ficolin-1 granule lumen, collagen binding), forming an ECM/immune microenvironment module. This network intuitively reveals that ivermectin may induce systemic cellular responses by simultaneously interfering with these two functional modules (**Figure 6C**). To further validate the expression regulation patterns of the core targets of ivermectin, transcriptomic data from esophageal squamous cell carcinoma cell lines treated with ivermectin (GSE201404) were obtained from the GEO database. The results showed that after ivermectin intervention, among the five core targets in both cell lines, the expression levels of PIK3CA and EGFR were consistently upregulated, while HSPG2, TGFB1, and ERBB2 were consistently downregulated. Moreover, the direction of expression changes of these targets exhibited a clearly modular pattern consistent with their functional assignments (**Figures 6D, E**). Notably, these findings also suggest that the regulation of these targets by ivermectin may be conserved to some extent and not limited to specific cell types.

### 3.10 Molecular docking of ivermectin to key toxicity targets

**Figure 7:**
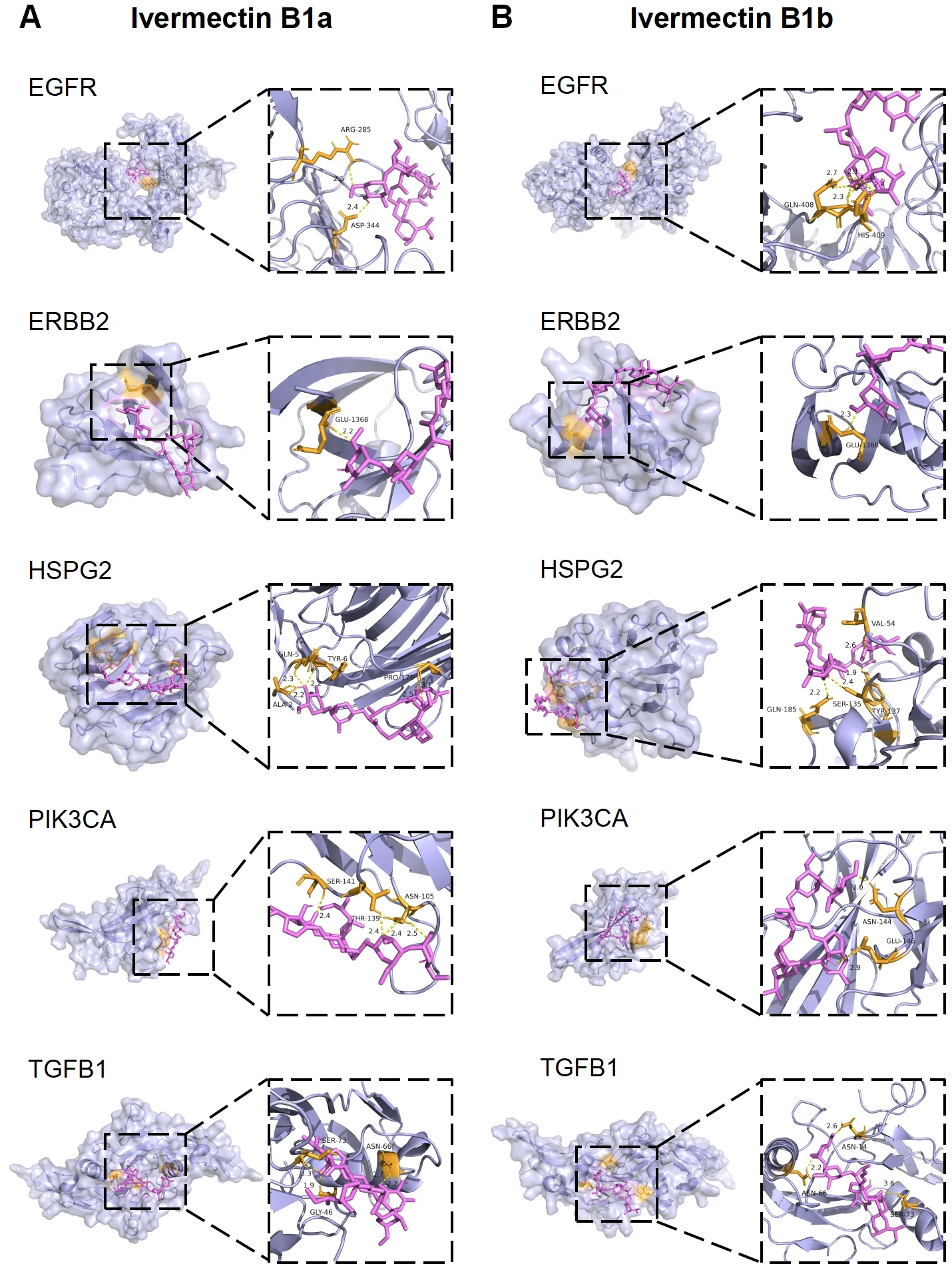
Diagram of molecular docking patterns between Ivermectin and key toxicity targets. **(A)** Binding site of Ivermectin B1a to proteins expressed by key toxicity genes. **(B)** Binding site of Ivermectin B1b to proteins expressed by key toxicity genes.

To further investigate the regulatory effects of ivermectin on these key toxicity targets, this study evaluated the direct binding affinity of the two main components of ivermectin (Ivermectin B1a and Ivermectin B1b) to the five core targets through molecular docking. The results showed that the binding energies of both components to all targets were below -7.0 kcal/mol, indicating that high-affinity spontaneous binding can occur. Among these, both components exhibited the lowest binding energies to EGFR (B1a: -8.7 kcal/mol; B1b: -10.0 kcal/mol), suggesting that EGFR is the most preferred molecular target of ivermectin (**Figures 7A, B**).

## 4 Discussion

### 4.1 Summary of key findings

As the clinical application scope of ivermectin expands to include potential antiviral, anti-inflammatory, and anti-tumor effects (Gandasegui et al., 2022; Patel et al., 2025; Shang et al., 2025), evaluating its comprehensive safety profile is imperative. This study integrated FAERS pharmacovigilance data, network toxicology, transcriptomic validation, molecular docking, and ADMET predictions to systematically investigate the adverse drug reactions (ADRs) of ivermectin and their toxicological mechanisms. We identified significant safety signals primarily in the eye, nervous system, and general disorders. In silico and transcriptomic analyses predicted five candidate targets (EGFR, ERBB2, TGFB1, PIK3CA, and HSPG2) involved in signal transduction and extracellular matrix remodeling, alongside identifying high theoretical risks for specific toxicities.

### 4.2 Comparison with existing literature

Our pharmacovigilance findings align with and extend previous clinical observations. At the PT level, common ADRs included asthenia, headache, and pyrexia, which are consistent with the known safety profile of ivermectin (López-Medina et al., 2021; Mikamo et al., 2024; Jalloh et al., 2026). The high signal intensity for eye disorders and nervous system disorders (including severe events like encephalopathy) is consistent with ivermectin’s known ability to cross blood barriers, facilitated by its high lipophilicity (Fobi et al., 2000; Edwards, 2003; Chandler, 2018; Opoku et al., 2018; Kanza et al., 2024; Rezaei et al., 2025). Notably, Ear and labyrinth disorders also exhibited a high signal intensity, highlighting the need for regular auditory monitoring during long-term ivermectin use (Little and Cosetti, 2021; Coffin et al., 2022). Furthermore, subgroup analysis revealed sex-specific differences (asthenia in males, headache in females), which align with documented variations in drug metabolism, hormone levels, or tissue sensitivity, suggesting a need for personalized monitoring (Moyer et al., 2019; Lakshmikanth et al., 2024).

It is also crucial to acknowledge the Mazzotti reaction, a severe immune-mediated inflammatory response triggered by the rapid death of microfilariae following ivermectin administration (Antonio et al., 2022). Clinically, this syndrome manifests with pyrexia, pruritus, erythema, and acute ocular inflammation, closely mirroring the prominent safety signals detected in our FAERS analysis. Consequently, relying solely on spontaneous reporting systems makes it challenging to definitively disentangle direct pharmacological toxicity from this parasite-death-driven immune cascade. However, our systemic target predictions suggest inherent off-target multiorgan liabilities exist alongside this immune response.

### 4.3 Mechanistic explanation

The observed multi-organ toxicities are potentially associated with the modulation of the predicted candidate targets. EGFR, a key receptor tyrosine kinase, exhibited the highest binding affinity with ivermectin. EGFR is widely expressed in various tissues, including the eye, nervous system, and skin, which aligns with the SOCs most affected by ivermectin ADRs (Domagala-Kulawik, 2019; Lu et al., 2026; Saw et al., 2026). Previous studies have linked EGFR dysregulation to neurotoxicity, ocular disorders, and skin reactions (Jayaswamy et al., 2023; Sanie-Jahromi et al., 2025; Nykaza et al., 2026). This direct interaction between ivermectin and EGFR is further supported by relevant research: one study demonstrated that ivermectin inhibits EGFR activation, thereby downregulating downstream pathways and interfering with normal signal transduction (Jiang et al., 2019). In addition to EGFR, ivermectin binds to HSP27, disrupting its chaperone function. Inhibiting HSP27’s cytoprotective effect increases cellular vulnerability and indirectly regulates EGFR signaling, forming a synergistic regulatory network that amplifies ivermectin’s toxic effects (Nappi et al., 2020).

These core targets participate in two primary functional modules: a signal transduction/growth regulation module (ERBB2, PIK3CA) and an ECM/immune microenvironment module (TGFB1, HSPG2) (Mendelsohn and Baselga, 2003; Hynes and Lane, 2005; Yarden and Pines, 2012). The concerted interference with these modules, structurally supported by molecular docking and validated by conserved transcriptomic alterations, provides a plausible biological basis for the systemic adverse reactions observed.

### 4.4 Strengths and limitations

A major strength of this study is the integration of real-world pharmacovigilance data with multi-omics methodologies to bridge the gap between clinical ADRs and preclinical molecular mechanisms (Cui et al., 2024; Sun et al., 2026). However, several limitations must be acknowledged. First, FAERS is a spontaneous reporting system; thus, the signals identified are not absolute risk estimates and cannot definitively establish causality due to potential underreporting and confounding by indication. Furthermore, spontaneous reporting databases frequently lack complete and standardized data regarding specific drug dosages, administration frequencies, and total duration of the dosing regimen. Because this critical clinical information is largely absent for the ivermectin cohorts, we were unable to conduct a dose-response analysis to determine whether the identified adverse events-particularly the severe neurological and ocular signals-are triggered by standard therapeutic doses or are the result of supratherapeutic exposure and toxicity.

Second, the in silico ADMET predictions require careful nuanced interpretation and contextualization within ivermectin’s established clinical profile. While the predicted high risk for skin sensitization aligns perfectly with the dermatological safety signals, such as pruritus and erythema, identified in our FAERS real-world data, ototoxicity and genotoxicity are not widely recognized as established clinical ADRs for ivermectin under standard therapeutic dosing. These discordant predictions may represent false positives, which is a recognized inherent limitation of machine learning-based ADMET algorithms when evaluating complex macrocyclic lactone structures. Alternatively, such toxicities might only manifest under extreme supratherapeutic exposure or accumulation. Furthermore, it must be acknowledged that the ADMETlab 3.0 platform provides these prediction scores as point estimates without confidence intervals or sensitivity analyses, limiting our ability to quantify the uncertainty of these specific risks. Therefore, while our ADMET results highlight potential theoretical liabilities that may warrant toxicological surveillance, clinical monitoring should primarily remain focused on the extensively documented neurological and ocular toxicities driven by ivermectin’s core pharmacological distribution.

Third, the transcriptomic validation in our study was exclusively performed using cancer cell lines (KYSE30 and KYSE70). We must acknowledge the inherent limitations of using cancer cell lines to study normal tissue toxicity, as oncogenic signaling pathways may not accurately reflect the physiological responses of healthy tissues. Despite extensive searches, no appropriate transcriptomic data from normal human tissues treated with ivermectin are currently available in public databases, which represents a major limitation of this study. However, based on publicly available normal tissue databases, including the GTEx portal, core targets exhibit baseline expression in relevant organs like the skin and central nervous system, providing a plausible biological basis for the observed off-target toxicities. Future studies must prioritize using primary normal cells or *in vivo* normal tissue models to validate these specific interactions dynamically.

Fourth, while our molecular docking simulations demonstrated favorable binding energies (≤ -7.0 kcal/mol) for the core targets, these findings remain predictive. Molecular docking does not intrinsically establish functional biological relevance (e.g., confirmation of kinase inhibition or activation), nor did we directly compare these binding energies against known endogenous ligands to definitively establish specificity (Zhu and Lv, 2025a; Zhu and Lv, 2025b). Therefore, our docking findings provide supportive evidence for potential target engagement, but must be interpreted with caution until validated by targeted *in vitro* functional assays.

### 4.5 Clinical implications

These findings provide critical insights for clinical practice. Clinicians should remain highly vigilant for primary neurological adverse events (such as toxic encephalopathy) and implement targeted monitoring for broader ocular and dermatological risks highlighted by these systemic molecular pathways. Furthermore, the manifestation of ivermectin-induced toxicity is heavily influenced by drug-drug interactions. Concomitant administration of ivermectin with strong CYP3A4 or P-glycoprotein inhibitors can drastically impair its metabolic clearance and facilitate unhindered penetration across the blood-brain barrie (Mtambo et al., 2024; Yilmaz et al., 2026). Clinicians must conduct rigorous reviews of concurrent medications and tailor safety monitoring strategies considering patient sex and baseline risk factors.

## 5 Conclusion

In summary, this study systematically elucidates the adverse drug reaction profile and underlying molecular mechanisms of ivermectin through an integrative approach combining pharmacovigilance, network toxicology, transcriptomic validation, molecular docking, and ADMET prediction. While ivermectin’s well-known GABAergic and glutamatergic mechanisms fundamentally underlie its severe neurotoxic side effects (such as encephalopathy), our network toxicology analysis suggests a complementary systemic mechanism potentially associated with multi-organ toxicity. Specifically, we identified five predicted candidate targets—EGFR, ERBB2, TGFB1, PIK3CA, and HSPG2—that may facilitate adverse reactions across ocular, nervous, and general systems. Clinicians must remain highly vigilant for primary neurological adverse events, while also implementing targeted monitoring for the broader ocular, ototoxic, and genotoxic risks highlighted by these systemic molecular pathways.

## Data Availability

The FDA Adverse Event Reporting System (FAERS) database (https://open.fda.gov/data/downloads/); PubChem database (https://pubchem.ncbi.nlm.nih.gov/); TOXRIC (https://toxric.bioinforai.tech/); Similarity Ensemble Approach (SEA, https://sea.bkslab.org/); ChEMBL (https://www.ebi.ac.uk/chembl/); Comparative Toxicogenomics Database (CTD, https://ctdbase.org/); TargetNet (http://targetnet.scbdd.com/); GeneCards (https://www.genecards.org/); SuperPRED (https://prediction.charite.de/); STRING database (https://cn.string-db.org/); RCSB PDB database (https://www.rcsb.org); ADMETlab 3.0 platform (https://admetlab3.scbdd.com/server/screening). The custom code and scripts used for data extraction and disproportionality analysis during the current study are available from the corresponding author upon reasonable request.
